# Elective home education of children with neurodevelopmental conditions before and after the COVID-19 pandemic started

**DOI:** 10.3389/fpsyg.2022.995217

**Published:** 2022-11-10

**Authors:** Laura Paulauskaite, Amanda Timmerman, Athanasia Kouroupa, Amanda Allard, Kylie M. Gray, Richard P. Hastings, David Heyne, Glenn A. Melvin, Bruce Tonge, Vasiliki Totsika

**Affiliations:** ^1^Social Research Institute, University College London, London, United Kingdom; ^2^Division of Psychiatry, University College London, London, United Kingdom; ^3^Council for Disabled Children, National Children’s Bureau, London, United Kingdom; ^4^Centre for Educational Development, Appraisal, and Research (CEDAR), University of Warwick, Coventry, United Kingdom; ^5^Department of Psychiatry, Monash University, Melbourne, VIC, Australia; ^6^Developmental and Educational Psychology Unit, Faculty of Social and Behavioural Sciences, Institute of Psychology, Leiden University, Leiden, Netherlands; ^7^School of Psychology, Faculty of Health, Deakin University, Geelong, VIC, Australia

**Keywords:** elective home education, intellectual disability, autism, mental health, COVID-19

## Abstract

COVID-19 brought disruptions to children’s education and mental health, and accelerated school de-registration rates. We investigated Elective Home Education (EHE) in families of children with a neurodevelopmental condition. A total of 158 parents of 5–15 year-old children with neurodevelopmental conditions (80% autistic) provided information on reasons for de-registration, their experience of EHE, and children’s mental health. Few differences were found between children participating in EHE before and after the pandemic started. Low satisfaction with school for not meeting children’s additional needs was the main reason for de-registering in both groups. COVID-19 had a more limited role in parents’ decision to de-register. The main advantage of EHE reported in both groups was the provision of personalised education and one-to-one support. Levels of anxiety, internalising and externalising problems were similar between children participating in EHE before and after the pandemic started, and also similar between all children in EHE and school-registered children (*N* = 1,079).

## Introduction

Elective Home Education (EHE) refers to the provision of education in the family’s home or a location outside of a school (e.g., online; Department for Education, 2019). When a child is home educated, parents take full responsibility for their child’s education and the associated costs (Department for Education, 2019). Children may be home educated for their whole education without ever attending a school, or they may be de-registered from school after a period of school education (Department for Education, 2019). Therefore, school deregistration and EHE may be linked to chronic school non-attendance (Schoeneberger, 2012).

The number of children in home education has been rising steadily in the UK and across the world in the past few years (Department for Education, 2019; Kunzman and Gaither, 2020). Recent data suggest that the pandemic led to further increases in EHE, particularly among families with children with a neurodevelopmental condition (The Association of Directors of Children’s Services, 2020, 2021). The present study investigates the experience of EHE in families of children with a neurodevelopmental condition (intellectual disability and/or autism), including in families who de-registered their child from school after the start of the COVID-19 pandemic in the United Kingdom.

In March 2019, there were 60,544 registered home educated children in England (about 0.7% of the whole student population), and it was estimated that numbers increased by 20% yearly for the 5 years before that (Department for Education, 2020; Office of the Schools Adjudicator, 2021). In the absence of a national register of home education, these numbers are likely an underestimate because parents are not obligated to report removing their children from school except when de-registering children with special educational needs and disabilities (SEND) - a term used in English educational settings to refer broadly to all children with any learning difficulty or/and disability that require additional support in schools (Department for Education, 2015).Research on reasons for de-registration from school points to parent dissatisfaction with school (including having issues with a teacher or/and other students), logistic reasons (such as moving to a different area), child mental or/and physical health problems, and religious or philosophical reasons (such as feeling that education at schools is too restrictive or formal; Smith and Nelson, 2015).

### Children with neurodevelopmental conditions, school attendance, and elective home education

Children with neurodevelopmental conditions such as intellectual disability or autism have complex needs (e.g., cognitive difficulties, limitations in social and verbal communication skills, and sensory processing issues) and many require individualised support and input from professionals for their learning and development (Buckley et al., 2020; Fleming et al., 2020). The prevalence of mental health problems in children with neurodevelopmental conditions is at least double that of typically developing children (Lai et al., 2019; Totsika et al., 2022). On average, their academic attainment is at the bottom of national indicators (Department for Education, 2014) and many of them feel isolated and/or are bullied at school (Humphrey and Hebron, 2015; Goodall, 2018; Bower, 2021). These children often experience problems with school attendance at rates higher than typically developing children (Munkhaugen et al., 2017; Black and Zablotsky, 2018; Ochi et al., 2020). Such problems are often a precursor to school de-registration and EHE (Munkhaugen et al., 2017; Black and Zablotsky, 2018; Ochi et al., 2020).

Research on reasons for EHE in children with neurodevelopmental conditions has been predominantly conducted with parents of autistic children (Arora, 2006; Kidd and Kaczmarek, 2010; Parsons and Lewis, 2010; Kendall and Taylor, 2016; O’Hagan et al., 2021). In these studies, the most frequently reported reason given by parents for providing EHE was that schools failed to meet the needs of their children. These failures included schools lacking knowledge and skills about how to educate children with complex needs and/or not providing education to match children’s needs (e.g., individualised, and flexible learning); children being bullied at school, experiencing mental health difficulties and/or refusing to attend (Kidd and Kaczmarek, 2010; Parsons and Lewis, 2010; Kendall and Taylor, 2016; O’Hagan et al., 2021). Some parents also reported that they felt pressure from schools to de-register their children from school and that EHE was rarely a “choice” but rather the only option (Kidd and Kaczmarek, 2010; Parsons and Lewis, 2010; O’Hagan et al., 2021).

### Elective home education and child mental health

The negative experiences of school attendance in some children (e.g., bullying, stressors associated with academic achievements or/and not receiving adequate support for learning) may be a contributing factor towards children’s poor mental health (Heyne et al., 2022). In contrast, home education is tailored to the child’s needs and avoids some of the environmental stressors associated with school (e.g., bullying; Maxwell et al., 2020).Therefore, it could be hypothesised that home education might be associated with better mental health.

Empirical evidence about the mental health of children participating in EHE is limited. The literature has predominantly compared the mental health of typically developing children participating in home education to that of school-registered peers, reporting mixed results (Guterman and Neuman, 2017; Schepis et al., 2020; Chen et al., 2021). First, researchers in Israel (Guterman and Neuman, 2017) compared depression scores, attachment security, and internalising and externalising problems of home educated children (*N* = 65, aged 6–12 years) to school-registered children (*N* = 101) matched on age, gender, religiosity, and family social economic circumstances. Findings showed that school-registered children had higher depression scores and externalising problems compared to home educated children. However, there was no difference between the groups on child internalising problems and attachment security (Guterman and Neuman, 2017). Second, findings from a national survey in the United States (Schepis et al., 2020) indicated a lower incidence of depression and lower levels of mental health treatment receipt among home educated adolescents (aged on average 14 years) compared to school-registered children. However, it is unclear if lower rates of treatments received indicated better mental health or more unfulfilled needs in this group. Third, a longitudinal study carried out in the U.S. (Chen et al., 2021) compared the mental health of home educated children to school-registered children at baseline (aged on average 14.56 years, range 11–19 years) and at 10-year follow-up (aged on average 25.10 years). At follow-up there were no significant differences in depression and anxiety between home educated and school-registered children but post-traumatic stress disorder (PTSD) symptoms were higher in home educated children. The difference in findings across the three studies could be due to sample differences. For example, Chen et al. (2021) used data from a nurses’ cohort and thus all parents in the study were highly educated. Differences may also be due to how children’s mental health outcomes were assessed; in Schepis et al. (2020) children’s mental health was assessed by mental health treatments received rather than standardised measures of child mental health symptoms.

There are no studies comparing the mental health of home educated children and school-registered children with neurodevelopmental conditions, despite the increased mental health needs in this population. A small number of qualitative studies have been mainly carried out with parents of children with SEND (Arora, 2006; Kidd and Kaczmarek, 2010; Parsons and Lewis, 2010; Kendall and Taylor, 2016; O’Hagan et al., 2021). In these studies, parents reported that after choosing to educate their children at home, their children appeared to be less anxious, happier, and more confident (Arora, 2006; Kidd and Kaczmarek, 2010; Parsons and Lewis, 2010; Kendall and Taylor, 2016; O’Hagan et al., 2021). Some parents reported an increase in children’s social skills and academic achievement, attributed to the fact that the education provided at home was flexible and individualised (Kidd and Kaczmarek, 2010; Parsons and Lewis, 2010; O’Hagan et al., 2021). Overall, findings from this limited number of small-scale qualitative studies suggest likely improvements in mental health.

### Elective home education accelerated during the pandemic

The COVID-19 pandemic is believed to have accelerated the rate of uptake of EHE in the United Kingdom. A survey carried out in October 2020 by the Association of Directors of Children’s Services (ADCS) collected data from local authorities in England on the number of all children in their area they believed were participating in EHE (The Association of Directors of Children’s Services, 2020). Data in these areas were only available from parents who volunteered such information and thus do not reflect the actual number. Findings from the survey suggested that since the start of the pandemic the number of children participating in EHE might have increased by 38% (The Association of Directors of Children’s Services, 2020). In that survey, local authorities reported that the most frequent reason provided by parents was health concerns associated with COVID-19 (The Association of Directors of Children’s Services, 2020). In a 2021 survey (The Association of Directors of Children’s Services, 2021), some local authorities reported that there had been a significant increase of children with SEND de-registered from school during the pandemic.

Overall, data appear to indicate an increase in the number of families opting for EHE and this increase appears to have accelerated after the COVID-19 pandemic where more families, including families of children with neurodevelopmental conditions, were de-registering their child from school (The Association of Directors of Children’s Services, 2020, 2021). This increase in de-registration may have been associated with health concerns due to COVID-19, although it is not known whether reasons for de-registering a child with neurodevelopmental conditions differed before and after the pandemic started. This information would provide useful insight in families’ decision making around de-registration as a result of COVID-19.

### COVID-19 disruptions to education and the mental health of children with neurodevelopmental conditions

The mental health of children with a neurodevelopmental condition deteriorated during the pandemic (Nonweiler et al., 2020; Guller et al., 2021; Masi et al., 2021). Educational disruptions experienced during the pandemic might have impacted on children’s mental health and families’ subsequent decision to de-register from school. Some evidence from data on home schooling (that is, the provision of education at home for a school-registered child while schools were closed during the pandemic) indicated mixed experiences in families of children with a neurodevelopmental condition (Greenway and Eaton-Thomas, 2020; Asbury et al., 2021; English, 2021; Ludgate et al., 2021; Wenham et al., 2021). Some parents reported that their children “thrived” during home schooling (Greenway and Eaton-Thomas, 2020; English, 2021; Ludgate et al., 2021). In Wenham et al. (2021) study parents reported that they were not considering sending their children back to school after the lockdowns were lifted in the United Kingdom and already de-registered their children from school because children’s well-being had improved since home schooling. Other parents reported that the child’s mental health had deteriorated during home schooling possibly due to loss of regular school support (Greenway and Eaton-Thomas, 2020; Asbury et al., 2021; Ludgate et al., 2021; Wenham et al., 2021). As these findings are from studies with school-registered children, they are not necessarily generalisable to children participating in EHE. Therefore, the impact that COVID-19 might have had on these children’s mental health has not been explored.

### The present study

The aim of the present study was to investigate EHE in UK families of children with neurodevelopmental conditions, namely autism and/or intellectual disability. We explored parents’ experience of EHE as well as reasons for school de-registration before and after the COVID-19 pandemic started. Further, we investigated, for the first-time, child mental health outcomes (anxiety, internalising, and externalising problems) both in relation to the timing of de-registration and in comparison with school-registered children.

## Materials and methods

### Procedure

Ethical approval was provided by the University College London Research Ethics Committee (Reference number: 20633/001). To be eligible to participate, parents had to have a 5 to 15-year-old child with a neurodevelopmental condition, namely autism and/or intellectual disability (and any co-occurring conditions) and be resident in any of the four UK countries. Eligible children could have been participating in EHE or registered with a school. The study included a total of 1,234 parents of 5- to 15-year-old children of whom 1,076 were parents of school-registered children. Participation in the survey was anonymous. Data were collected through an online survey. The focus of the survey was on the educational experiences of children with neurodevelopmental conditions 1 year after the start of COVID-19 in the United Kingdom. Data were collected between June and November 2021. Parents were invited to take part *via* social media posts (e.g., Twitter), mailing lists and newsletters by the study team and third sector recruitment partners (e.g., charities for children with neurodevelopmental conditions and EHE support groups). A Parent Advisory Group guided all stages of the study including survey development, data analysis and interpretation.

### Participants

Participants were 158 parents of home educated 5–15 year-old children. Among the 158 parents of home educated children, 93 parents had children participating in EHE before March 2020 (pre-pandemic EHE group) while 65 parents had children de-registered from school after the pandemic started in the UK in March 2020 (pandemic EHE group). Among the pre-pandemic EHE group, 23 children had always been participating in EHE meaning they had never registered with a school, and 68 children were de-registered at some point pre-pandemic (information was missing for 2 children in this group).

Table 1 reports participants’ demographic characteristics. In both groups, the majority of children were boys (60.0% in EHE pre-pandemic and 69.2% in EHE pandemic) aged on average 11 years-old (Mean age = 11 years, SD = 2.9, range = 5–15 years and Mean age = 10.9 years, SD = 2.7, range = 5–15 years, respectively for the EHE pre-pandemic group and EHE pandemic group). Most children were autistic: 82.6% in EHE pre-pandemic group and 76.9% in the EHE pandemic group. The majority of children lived in England (72.8% in EHE pre-pandemic and 78.1% EHE pandemic). About a third of children in both EHE groups had intellectual disability (28.3% in EHE pre-pandemic group and 26.2% in EHE pandemic group). Children were very similar in terms of their profile with some exceptions: there was a higher proportion of White ethnicity in the EHE pre-pandemic group, whereas more children in the EHE pandemic group had a formal recognition of their special educational needs (e.g., a SEND plan). Please see Table 1 for more details.

**Table 1:** Participants’ demographic characteristics.

Total EHE sample	EHE pre-pandemic	EHE pandemic
*N* = 158	*N* = 93	*N* = 65	*p* value
Child characteristics	*N* (%)	*N* (%)		*N* (%)
Child is a boy	101 (63.9%)	56 (60.9%)	45 (69.2%)	0.28
Child age in years (SD)	11 (2.8)	11 (2.9)	10.9 (2.7)	0.83
Ethnicity – White	148 (93.7%)	90 (97.8%)	57 (87.7%)	0.01*
Lives in England	118 (75.2%)	67 (72.8%)	50 (78.1%)	0.45
Lives with family full-time	154 (98.1%)	90 (97.8%)	63 (98.4%)	0.78
Lives with two or more parents	120 (76.4%)	68 (73.9%)	51 (79.7%)	0.4
**Neurodevelopmental conditions**				
Child has ID	44 (27.9%)	26 (28.3%)	17 (26.2%)	0.77
Child has ASD	126 (79.8%)	76 (82.6%)	50 (76.9%)	0.38
Has two or more NDCs	72 (46.8%)	46 (51.1%)	26 (41.3%)	0.23
**Additional health problems**				
Deaf or blind	10 (6.3%)	4 (4.4%)	6 (9.2%)	0.22
Mobility problems	22 (13.9%)	14 (15.2%)	8 (12.3%)	0.61
Physical health problems	34 (21.5%)	20 (21.7%)	14 (21.5%)	0.97
Clinically extremely vulnerable*	9 (5.8%)	5 (5.5%)	4 (6.3%)	0.84
Shielding due to COVID-19*	13 (8.3%)	8 (8.8%)	5 (7.8%)	0.83
**Formal recognition of special educational needs**				
Has SEND plan	69 (43.7%)	33 (35.9%)	35 (53.9%)	0.02*
**Parent characteristics**				
Respondent is mother	145 (92.4%)	85 (92.4%)	59 (92.2%)	0.97
Respondent age (SD)	44.2 (8.1)	44.4 (8.0)	43.8 (8.3)	0.66
Mean N of children (SD)	2.1 (1.1)	2 (1.1)	2 (1.2)	0.97
Single parent household	37 (23.6%)	24 (26.1%)	13 (20.3%)	0.38
Disability	66 (45.8%)	38 (45.8%)	28 (45.9%)	0.99
Clinically extremely vulnerable*	14 (9.7%)	6 (7.2%)	8 (13.1%)	0.24
Educated at university degree level	76 (53.5%)	47 (57.3%)	29 (48.3%)	0.29
At least one parent is employed	107 (67.7%)	62 (67.4%)	45 (69.2%)	0.8
Struggling financially	31 (19.6%)	22 (23.9%)	9 (13.9%)	0.12
Socioeconomic deprivation (SD)	1.6 (0.9)	1.7 (0.9)	1.5 (0.8)	0.15

*Significant results; EHE = Elective Home Education; SD = standard deviation; ID = intellectual disability; ASD = autism spectrum disorder; NDCs = neurodevelopmental conditions; SEND = special educational needs and disability, clinically extremely vulnerable = classification defined by the UK Department of Health and Social Care (Department of Health and Social Care, 2020); shielding due to COVID-19 = staying at home avoiding contact with other people (Department of Health and Social Care, 2020).

Similarly, families’ profiles were very similar across both groups, with the majority of respondents being mothers (92.4% in the EHE pre-pandemic and 92.2% in the EHE pandemic group) aged on average 44 years-old (Mean age = 44.4 years, SD = 8, range = 27–60 years and Mean age = 43.8 years, SD = 8.3, range = 29–66 years, respectively, for both groups). Across the EHE groups, similar numbers of parents reported having a disability (45.8 and 45.9% and, respectively, for two groups) and having at least one parent employed in the household (67.4 and 69.2%, respectively, for two groups). Whilst non-significant, a higher percentage of families in the EHE pre-pandemic group reported being single parent families (26.1%), being educated to a university degree level (67.4%) and experiencing financial struggles (23.9%). It is worth noting that non-significant differences between the EHE pre-pandemic group and EHE pandemic group might be due small sample sizes and the reduced power to detect significant differences (see Table 1).

### Measures

#### Elective home education

Parents indicated whether their child participated in EHE in May 2021 (yes/no). If not, parents were then asked whether their child was registered to attend school in March 2020 (the month the COVID-19 pandemic started in the UK). Parents who indicated their child was de-registered from school in May 2021 and March 2020 formed the EHE pre-pandemic group and were subsequently asked whether their child was ever registered with a school (yes/no) as well as the month and year of school de-registration. Parents who indicated that their child was de-registered from school in May 2021 but was still registered with a school in March 2020 formed the EHE pandemic group and were subsequently asked to indicate the month of de-registration starting from March 2020 until the time of survey completion.

##### Reasons for de-registration

All parents, except for those whose child had always been participating in EHE, were asked to indicate reasons for de-registration out of a list of 11 possible reasons (see Table 2). Reasons for de-registration were identified from evidence from existing studies (see Introduction) and reviewed by the Parent Advisory Group (see Procedure) for completeness and relevance to this population. Parents could select all relevant reasons. All parents of children participating in EHE were asked to indicate whether they were currently awaiting a school place (yes/no).

**Table 2:** Reasons for school de-registration.

Reasons	EHE pre-pandemic *N* = 68	EHE pandemic *N* = 63
I did not feel my child was safe from COVID-19 at school	N/A	15 (23.8%)
I did not feel the school provided a good education to my child	30 (44.1%)	30 (47.6%)
My child was unhappy at the school	50 (73.5%)	38 (60.3%)
My child did not want to go to that school	32 (47.1%)	29 (46%)
My child’s mental health had deteriorated	52 (76.5%)	35 (55.5%)
I felt that could provide a better education for my child at home	36 (52.9%)	44 (69.8%)
My child’s additional needs were not met sufficiently in the school	53 (77.9%)	45 (71.4%)
The school had off rolled my child*	4 (5.8%)	4 (6.3%)
The school told me that my child was at risk of exclusion	4 (5.8%)	5 (7.9%)
The school had permanently excluded my child	N/A	N/A
I felt pressured from the school to remove my child	6 (8.8%)	6 (9.5%)
Other, please describe	21 (30.9%)	26 (41.3%)

EHE = Elective Home Education; * the school had off rolled my child: informal school exclusion.

##### Support for learning and equipment needed for elective home education

Data were collected on the frequency that different providers were supporting the child’s home education. Providers included the participating parent (“I teach or support my child with their learning”), another parent, a sibling, a private tutor, or an online group. Parents indicated if support was provided daily, weekly (several times or once a week), monthly or less frequently. A list of equipment items (e.g., computer, desk, and internet) was provided for parents to indicate if they had access to it, if they did not have access to it but needed it, or if they had access to it but needed/wanted better quality.

##### Parents’ satisfaction with elective home education

Parents were asked to indicate on a 1–10 scale their level of satisfaction with EHE, with 1 being “extremely dissatisfied” to 10 being “extremely satisfied.”

##### Barriers and facilitators of elective home education

To understand parents’ experiences of EHE we asked them to write up to three barriers and up to three facilitators of EHE in free-text boxes in the survey.

#### Child mental health

##### Anxiety

Parents were asked to complete the anxiety subscale of the Developmental Behaviour Checklist – Parent Report (DBC2) to collect information on child anxiety symptoms (Gray et al., 2018). The DBC2 was selected because it was developed specifically for children with neurodevelopmental conditions, such as intellectual disability and autism, and has good psychometric properties (Einfeld and Tonge, 1995; Gray et al., 2018). The Anxiety scale includes 12 items asking parents to rate their children’s behaviour over the last 6 months on a 3-point scale (“not true as far as I know or not applicable to my child,” “somewhat true or sometimes true” and “often true or very true”; scoring: 0–2). A total anxiety score was calculated by adding responses from all 12 questions (range: 0–24) with higher scores indicating higher levels of anxiety problems. The Cronbach’s alpha for the Anxiety subscale of the total EHE sample was 0.77 indicating good internal consistency.

##### Internalising and externalising problems

Parents were asked to complete the parent version of the Strengths and Difficulties Questionnaire (SDQ; Goodman, 1997) to collect information on child internalising and externalising problems. The SDQ has been widely used to assess emotional and behavioural problems in typically developing young people and is also a reliable measure to use with children and young people with intellectual disability (Murray et al., 2021). The SDQ has 25 questions, five in each of the five subscales: Emotional Problems, Conduct Problems, Hyperactivity, Peer Problems and Prosocial Behaviour. In this study, parents were presented with all 25 questions and asked to rate their child’s behaviour over the last 6 months on a 3-point scale (“not true,” “somewhat true” and “certainly true”; scoring: 0–2). The Cronbach’s alpha for the internalising emotional problems of the total EHE sample was 0.69 indicating acceptable internal consistency. A total score of child internalising problems was calculated by adding scores of two subscales: Emotional Problems and Peer Problems (range of scores: 0–20). A total score of child externalising problems was calculated by adding scores of two subscales: Conduct Problems and Hyperactivity (range of scores: 0–20). The Cronbach’s alpha for the externalising emotional problems of the total EHE sample was 0.77 indicating good internal consistency.

#### Demographic information

We collected demographic information on parents’ gender, age, relationship to the child, educational qualification, employment status, disability status and whether they were clinically extremely vulnerable to the COVID-19 infection as determined by the UK Department of Health (Department of Health and Social Care, 2020). Data were also collected on child age, gender, ethnicity, neurodevelopmental and health conditions, formal recognition status of special education needs (e.g., whether a child had a SEND plan), country of residence. A measure of subjective poverty assessed whether families felt were struggling financially: “How well would you say your family is managing financially these days? Would you say you are..?.” The variable is rated on a 5-point scale (living comfortably, doing alright, just about getting by, finding it quite difficult, finding it very difficult). The last two options on this scale were combined to indicate the experience of subjective poverty in a family. A family socioeconomic deprivation variable was created by combining information on four dichotomised indicators: subjective poverty (struggling financially/managing OK), level of parent educational qualification (above/below university degree level), employment (at least one adult employed in household/ unemployed), and single parent household (one parent/carer in the household/more than one).

### Data analysis

#### Quantitative analyses

We used STATA version 17 to analyse quantitative data. We compared demographic characteristics of EHE pre-pandemic and EHE pandemic groups using t-tests for continuous variables and chi-square tests for categorical variables. We report descriptive statistics for reasons for de-registration, practical arrangements, and parents’ satisfaction with EHE. Satisfaction with EHE was compared between the two EHE groups (independent t-test). We compared mental health levels (anxiety, internalising and externalising problems) between the two EHE groups using independent t-tests. Mental health levels were also compared between all EHE participants (N = 158) and school-registered children (N = 1,076). Comparisons were initially unadjusted (t-tests) to examine whether differences in mental health outcomes were present between groups. Comparisons were then adjusted for a range of potential confounding variables (in linear regression models) to investigate whether group differences would be attenuated after controlling for other variables known to be associated with child mental health. To identify eligible confounders, we drew on relevant literature that examined mental health outcomes of home educated children in the general population (Guterman and Neuman, 2017; Schepis et al., 2020; Chen et al., 2021) as well as theoretical models of correlates of school attendance problems (Melvin et al., 2019).We proceeded to examine the univariate associations between likely covariates (child’s age, child’s gender, ethnicity, country where the child lives, the presence of additional physical health conditions, the presence of two or more neurodevelopmental conditions, the presence of intellectual disability, having formal recognition of special educational needs, family socioeconomic deprivation and parent disability) and each of the three child mental health outcomes (see Supplementary material 1 for findings). We adopted a parsimonious approach to model building and included in the final model variables significantly associated with each child mental health outcome: the presence of additional physical health problems, the presence of two or more neurodevelopmental conditions, the presence of intellectual disability, having formal recognition of special educational needs, family socioeconomic deprivation and parent disability.

#### Qualitative analysis

Content analysis (Bengtsson, 2016) was performed to analyse qualitative data on barriers and facilitators of home learning in the two EHE groups using NVivo 2020. Content analysis allows for a bottom-up coding of the data which was consistent with the aims of the study; no *a priori* assumptions were made about likely barriers and facilitators in this group of participants. Data were coded following a bottom-up approach in each group independently and researchers then examined whether themes identified in each group were similar or different. The themes identified were the same between the two groups and we then proceeded to investigate the frequency of the theme within each group. Content analysis uses both qualitative and quantitative methodology (i.e., examines the frequency each theme was reported within the data set) and can be used inductively by analysing what emerges from the data (Bengtsson, 2016). The presence of two groups in our study who experienced EHE at a different point in time as well as the likely impact of COVID-19 in their experience and decision indicated the need to compare themes across the qualitative data.

After thorough familiarisation with the data, two researchers (AT and LP) developed the codebook for analysing the data which was shared for discussion with the study team and the Parent Advisory Group. The codebook development involved initially reading the data and developing codes inductively by two researchers independently. Then, the researchers worked together to finalise the coding scheme which involved merging the codes that were semantically related, re-naming the codes, providing descriptions to the codes using participants’ quotes and grouping semantically related codes into bigger categories-themes. One researcher (AT) coded all the data and another researcher (LP) coded 20% of the data independently for an inter-rater reliability assessment. The agreement between researchers was very good (Cohen’s Kappa was 0.81) based on parameters suggested by Landis and Koch (1977). Data were coded separately for each EHE group. Below we report the frequency of reported barriers and facilitators calculated by dividing the number of mentions (within each group) by the overall number of barriers or facilitators reported.

## Results

Children in the EHE pandemic group were de-registered from school any time between March 2020 and September 2021 (with 25.4% of children in this EHE group deregistering in September 2020; see Supplementary material 2). Children participating in EHE pre-pandemic were de-registered from school between 2009 and up to 2020, but before March 2020 (with 32.3% reporting de-registering in 2019; see Supplementary material 2).

Reasons for school de-registration as selected by parents are shown in Table 2. The most frequent reason for de-registering pre-pandemic was that the child’s additional needs were not met sufficiently in the school (77.9%) followed by the child’s mental health deterioration (76.5%) and the child being unhappy at school (73.5%). Twenty-one parents (30.9%) provided additional information on reasons for de-registration, including safeguarding risks/issues at the school without specifying what specific issues were (n = 6), bullying in the school (n = 6), and providing more detailed descriptions of the reasons specified in the table. The most frequent reasons for de-registering after the pandemic started were that the child’s additional needs were not met sufficiently in the school (71.4%) and that parents felt they could provide a better education at home (69.8%). Only 15 parents (23.8%) reported de-registering because they felt that the child was not safe from COVID-19 at school. Twenty-six parents (41.3%) provided additional free-text information on reasons for de-registration, including moving home (n = 3) and bullying in the school (n = 2).

Daily support for learning was provided by the responding parent in 84.1% of cases for children participating in EHE pre-pandemic while this was the case in 68.3% of families in EHE pandemic group. Siblings or other family members supported the child’s learning less than once per month. A private tutor and online teaching programmes were used to support child’s learning at home several times a week in both groups (Table 3). Parents most frequently reported that they needed but did not have access to special software (39.4% in the EHE pre-pandemic group and 38.1% in the EHE pandemic group) and other specialist equipment, e.g., books (32.1 and 28.1%, respectively, for both EHE groups, see Table 4).

**Table 3:** Type and frequency of support provided for child’s learning at home presented for two EHE groups.

	EHE pre-pandemic					EHE pandemic				
	Daily	Several times a week	Once a week	Monthly	Less often	Daily	Several times a week	Once a week	Monthly	Less often
Parent	74 (84.1%)	10 (11.4%)	1 (1.1%)	-	3 (3.4%)	43 (68.3%)	13 (20.6%)	2 (3.2%)	1 (1.6%)	4 (6.4%)
Family member	16 (19.1%)	23 (27.4%)	9 (10.7%)	3(3.6%)	33 (39.3%)	9 (16.1%)	16 (28.6%)	6 (10.7%)	2 (3.6%)	23 (41.1%)
Sibling	5 (7.3%)	7 (10.1%)	4 (5.8%)	2 (2.9%)	51 (73.9%)	2 (4.2%)	2 (4.2%)	5 (10.4%)	2 (4.2%)	37 (77.1%)
Private tutor	2 (2.7%)	16 (21.3%)	14 (18.7%)	-	43 (57.3%)	1 (2%)	11(22.5%)	10 (20.4%)	1 (2%)	26 (53.1%)
Online school	2 (2.9%)	16 (23.2%)	11 (15.9%)	-	40 (58%)	6 (11.3%)	13 (24.5%)	4 (7.6%)	2 (3.8%)	28 (52.8%)

EHE = Elective Home Education.

**Table 4:** Type of equipment needed for child’s home learning presented for the two EHE groups.

Type of equipment	EHE pre-pandemic			EHE pandemic		
	Needed and had access to	Needed but did not have access	Had access but need more or better quality	Needed and had access to	Needed but did not have access	Had access but need more or better quality
Laptop, PC, or tablet	71 (82.6%)	4 (4.7%)	11 (12.8%)	46 (74.2%)	6 (9.7%)	10 (16.1%)
Smart phone	57 (86.4%)	2 (3%)	7 (10.6%)	44 (84.6%)	1 (1.9%)	7 (13.5%)
Printer	71 (84.5%)	3 (3.6%)	10 (11.9%)	48 (81.4%)	6 (10.2%)	5 (8.5%)
Internet access/data	79 (94.1%)	1 (1.2%)	4 (4.8%)	55 (94.8%)	1 (1.7%)	2 (3.5%)
Headphones	63 (86.3%)	3 (4.1%)	7 (9.6%)	41 (78.9%)	7 (13.5%)	4 (7.7%)
Special software	31 (43.7%)	28 (39.4%)	12 (16.9%)	19 (45.2%)	16 (38.1%)	7 (16.7%)
Webcam	50 (72.5%)	15 (21.7%)	4 (5.8%)	37 (78.7%)	6 (12.7%)	4 (8.5%)
Desk/table	69 (88.5%)	4 (5.1%)	5 (6.4%)	47 (82.5%)	7 (12.3%)	3 (5.3%)
Specialist equipment	38 (48.7%)	25 (32.1%)	15 (19.2%)	37 (64.9%)	16 (28.1%)	4 (7%)

EHE = Elective Home Education; PC = personal computer.

### Parents’ experience and satisfaction with EHE

Parents in both EHE groups reported being highly satisfied with EHE and there was no statistical difference between groups (Mean satisfaction score = 8.4 points, SD = 2 in the EHE pre-pandemic group and Mean satisfaction score = 8.0 points, SD = 2.3 in the EHE pandemic group; *t*(139) = −0.43, *p* = 0.24). Eight parents (12.3%) of children in the EHE pandemic group indicated that they were waiting for a place at a different school compared to three parents (3.4%) in the EHE pre-pandemic group.

Table 5 shows the barriers and facilitators of home education. Overall, similar barriers of home education were reported by parents of children in two EHE groups. The most frequently reported barrier of home education in both EHE groups was competing demands (30% of the barriers in EHE pre-pandemic and 43% of the barriers in EHE pandemic) followed by difficulties experienced due to child’s needs (20% of the barriers in EHE pre-pandemic and 21% of the barriers in EHE pandemic). It should be noted that nine parents (three in EHE pre-pandemic and six in EHE pandemic) reported that they had not experienced any barriers with EHE.

**Table 5:** Barriers and facilitators of Elective Home Education as reported by parents in this study.

Barriers				
Frequency of reporting	
Theme	Definition	Examples
			EHE pre-pandemic	EHE pandemic
30%	43%	Competing demands	The competing functions of EHE and the family impact on each other in ways that present a challenge. These include but are not limited to financial impact, other family commitments, the needs of parents, and difficulties with routines.	“Loss of income” “Being with child most of time/no time for yourself” “Caring for another member”
6%	8%	Home being unsuitable environment for learning	Aspects of the home environment make EHE more difficult, e.g., distractions or/and lack of space.	“Home distractions” “Access to TV and PlayStation have to be monitored and controlled” “Small, shared space and husband is working from home”
22%	15%	Difficulties accessing resources to support child learning	Families have limited access to resources that are typically available in schools including learning/assessment materials, sports/social activities, teachers/tutors and the internet. Also, the COVID-19 related difficulties accessing resources.	“Lack of access to exams” “Slow internet” “No access to social groups due to COVID”
21%	20%	Difficulties due to child’s needs	The child’s needs in terms of physical and mental health, behavioural issues, or learning problems make EHE more challenging.	“Child’s low attention span” “My daughter’s ADHD” “Child refusing, finds writing very stressful”
21%	15%	Lack of support or understanding from others	The parent feels lack of support or understanding from others, e.g., schools, local authorities (LAs), professionals, community, family, friends including difficulty getting helpful advice and guidance to improve their EHE delivery.	“Being forced into it with no apology from school” “Do not yet have access to therapies in EHCP as LA is refusing to help - we are at SEN Tribunal”
Facilitators				
**Frequency of reporting**		**Theme**	**Definition**	**Examples**
**EHE pre-pandemic**	**EHE pandemic**			
10%	14%	Availability of family’s own resources	The family’s own resources facilitate home learning in terms of physical home environment, skills, and family’s social capital (e.g., having supportive friends and family).	“My education level (PhD)” “Being an educator myself” “Being able to work from home” “Access to friends and family”
19%	22%	Availability of external resources	The family has access to external resources such as online and physical resources, the internet, tutors, places to go, and activities to take part that support child’s learning and development.	“Countless free resources” “Reduced entry to things like museums with disability living allowance” “Being able to meet up to learn in groups”
48%	39%	Able to provide personalised education experience	The family is able to provide flexible and personalised education that is adapted it to the child’s interests and needs.	“Not having to follow the curriculum” “Flexible learning” “One-to-one support which wasn’t available in school”
8%	7%	Child’s well-being is supported at home	The child’s well-being in terms of physical health, mental health, behavioural problems is good at home.	“No bullying” “My children are happy and thriving” “My daughter feels safe at home with less sensory noise and is able to learn better”
16%	14%	Good external support	Support is provided to the parent by people or organisations external to the family, including schools and teachers, Local Authorities (LAs), EHE support groups, and other professionals such as GPs, clinicians.	“School were supportive and even lent us materials” “Excellent support from local and national home ed. community” “Supportive professionals”

EHE = elective home education.

Facilitators of home education were similar between the two EHE groups. The most frequently reported facilitator in both EHE groups was being able to provide personalised education (48% of the facilitators in EHE pre-pandemic and 39% of the facilitators in EHE pandemic). Parents reported that having the freedom to personalise and tailor education to the child’s needs and interests as well as providing one to one support to the child facilitated their child’s learning at home. The second most frequently reported facilitator across both EHE groups was the availability of external resources (19% of the facilitators in EHE pre-pandemic and 20% of the facilitators in EHE pandemic). Parents reported that having access to external resources such as free online courses, books, the internet, private tutors, and activities in a community were supporting and facilitating their child’s learning and development.

### Child mental health outcomes

#### Anxiety

There was no statistical difference in DBC2 anxiety scores between children participating in EHE before and after the pandemic started [*t*(154) = 0.51, *p* = 0.51]. There was no statistical difference in child anxiety scores (DBC2 scores) between the total EHE group and the school-registered children [*t*(1168) = −0.25, *p* = 0.57] (Table 6). After adjusting for child covariates, family socioeconomic deprivation, and parent disability, there was still no difference in anxiety scores between the total sample of children participating in EHE and school-registered children (adjusted mean difference: 0.14 points, 95% CI: −0.87 to 0.90, *p* = 0.98).

**Table 6:** Child mental health outcomes between the total EHE sample and school-registered children.

	Total EHE sample	School-registered children	T-test coefficient (95% CI) *p* values	Regression coefficient (95% CI) *p* values
Outcome
	Mean (SD)	Mean (SD)	Unadjusted*	Adjusted*
DBC2 anxiety scores	12.56 (0.16)	12.80 (0.37)	−0.25 (CI: −1.09 to 0.60) *p* = 0.57	0.01 (CI: −0.8 to 0.90) *p* = 0.98
SDQ internalising problems score	11.22 (0.13)	11.46 (0.30)	−0.23 (CI: −0.91 to 0.43) *p* = 0.49	−0.28 (CI: −1.01 to 0.45) *p* = 0.45
SDQ externalising problems score	10.62 (0.12)	11.31 (0.31)	0.69 (CI: 0.06 to 1.32) *p* = 0.03*	−0.54 (CI: −1.20 to 0.12) *p* = 0.11

CI = confidence intervals; * = significant results; unadjusted* = *t*-tests; adjusted* = linear regression adjusted for child’s age, child’s gender, child’s ethnicity, child living in England, child having additional physical conditions, child having two or more neurodevelopmental conditions, child having intellectual disability, child having formal recognition of special educational needs and family socioeconomic deprivation and parent disability.

#### Internalising problems

There was no statistical difference in scores of internalising problems (SDQ scores) between children participating in EHE before and after the pandemic started [*t*(154) = −0.01, *p* = 0.99]. There was also no difference in internalising problems between the total EHE sample and the school-registered children [*t*(1136) = −0.23, *p* = 0.49]. After adjusting for child covariates, family socioeconomic deprivation, and parent disability, there was still no difference in levels of internalising problems between the total EHE sample and school-registered children (adjusted difference = −0.28 points, 95% CI: −1.01 to 0.44, *p* = 0.45).

#### Externalising problems

There was no evidence of a statistical difference in externalising problem levels between children participating in EHE before and after the pandemic started *(t*(154) = − 0.37, *p* = 0.55). There was weak evidence that scores of externalising problems were statistically higher in the school-registered children than in the total EHE sample (*p* = 0.03). The unadjusted SDQ score of externalising problems was 0.69 points higher (95% CI = 0.06 to 1.32) in school-registered children compared to the total EHE sample. However, after adjusting for child covariates, family socioeconomic deprivation, and parent disability variables, there was no evidence of a statistically significant difference between the total EHE sample and school-registered children on levels of externalising problems (adjusted difference = −0.54 points, 95% CI: −1.20 to 0.12, *p* = 0.11).

## Discussion

Overall, there were few differences between the children participating in EHE before and those participating in EHE after the pandemic. Parents’ reasons for de-registering their child from school before and after the pandemic were broadly similar. Interestingly, health concerns due to COVID-19 were not the main reason for de-registration during the pandemic; fewer than 24% of parents whose child was de-registered after the pandemic selected this as the reason for de-registration. This finding contrasts to the 2020 and 2021 EHE surveys in England (The Association of Directors of Children’s Services, 2020, 2021) where local authorities nominated health concerns due to COVID-19 as the main reason for parents selecting to de-register their children. Except for the fact these surveys were not restricted to neurodevelopmental conditions, it is also worth noting that data were not collected directly from parents. Differences in the target population and the survey design may explain the differences seen in the reasons reported.

Findings on parents’ top reasons for de-registration suggest an overall dissatisfaction with the school’s capacity for meeting the additional or different learning needs of these children as well as their mental health needs. For both EHE groups in our study, the most frequent reason for school de-registration was that the child’s additional needs were not met sufficiently in school. Our qualitative findings appear to confirm these findings; the main advantage of EHE, as experienced by both groups of parents, was the ability to provide personalised education and one to one support that their child was not receiving at a school. Our findings align with evidence on the educational experiences of school-registered children with neurodevelopmental conditions (Brede et al., 2017; Sproston et al., 2017; Anderson, 2020), and with the reasons for choosing home education reported by parents of children with SEND in the studies carried out before the COVID-19 pandemic (Arora, 2006; Kidd and Kaczmarek, 2010; Parsons and Lewis, 2010; Kendall and Taylor, 2016; O’Hagan et al., 2021). Taken together, the choice of EHE in families of children with a neurodevelopmental condition may be associated more strongly with perceived unmet learning and mental health needs in school; this association does not appear to have been disrupted by COVID-19, though it may have been compounded (Asbury et al., 2021).

EHE appeared to be working well for participating families. High levels of satisfaction with EHE were reported, and this was similar across groups. Families were mostly well equipped to support EHE at home both in terms of practical equipment and also support for learning. Support for learning was provided mostly by mothers (though substantially more so in the EHE pre-pandemic group), while others (other family members and tutors) supported the child on a weekly basis. Parents reported that managing competing demands (e.g., being a mother and an educator at the same time) and supporting the child’s complex needs (e.g., behavioural or additional difficulties) were the main difficulties of EHE. These findings echo parents’ experiences of providing home education to their children with SEND (Arora, 2006; Kidd and Kaczmarek, 2010; Parsons and Lewis, 2010; Kendall and Taylor, 2016; O’Hagan et al., 2021). On the other hand, parents’ perception that EHE’s affordance of individualised learning as the main facilitator of EHE might mean that the difficulties of providing EHE (e.g., managing competing demands, loss of income and less free time for themselves) might feel manageable considering the main benefit they see in their child.

We found no evidence of a statistically significant difference in levels of child mental health problems between children who were de-registered before the pandemic and those participating in EHE after the COVID-19 pandemic started. Overall, the two groups of children participating in EHE presented with almost identical levels of anxiety, internalising, and externalising problems. A recent systematic review that summarised evidence from the start of the pandemic (2020) concluded that children, in particular those with neurodevelopmental conditions, experienced an increase in anxiety and internalising symptoms following the start of the pandemic, though the evidence came mostly from studies without longitudinal data (Samji et al., 2022). In our study, where again we were unable to control for mental health levels prior to the pandemic, we found no evidence of worse mental health among children who were de-registered from school after the pandemic. However, deterioration in child’s mental health was more highly endorsed by parents as a reason for selecting home education prior to the pandemic.

We also found no evidence of a difference in mental health problems between children participating in EHE and school-registered children. While to date no previous studies compared the mental health of children with neurodevelopmental conditions between home education and school education, studies that did this with typically developing children produced mixed evidence (Guterman and Neuman, 2017; Schepis et al., 2020; Chen et al., 2021), with some finding differences in some aspects of mental health and other studies finding no differences. Better child mental health was perceived to be a facilitator of EHE by families in our study, but its occurrence was rather limited; only 7–8% of reported facilitators were about improved child well-being. Clearly, more research is needed to compare the mental health of children with neurodevelopmental conditions between the two educational settings, the school and the home. Additionally, future research needs to focus on academic outcomes of this group of children both because this is an area of great need but also because the main reason for choosing EHE as well as the main benefit of EHE appear to be the adaptation of the learning environment to suit the child’s different or additional learning needs.

### Strengths and limitations

This was the first study to explore EHE specifically in children with neurodevelopmental conditions in a sample much larger than previous studies (i.e., 158 participants). Participants were drawn from across all four United Kingdom nations, though the majority lived in England. The findings need to be interpreted while considering the study’s limitations. Data on children’s mental health were parent-reported and may not represent the actual levels of mental health problems experienced by their children. Future studies should seek the views of children with neurodevelopmental conditions on receiving EHE in addition to parent reports. Further, while our sample was larger than existing studies, it was still a small group compared to the likely overall population of children with neurodevelopmental conditions on EHE. Comparisons between children in EHE (*N* = 158) and school-registered children (*N* = 1,076) relied on unbalanced groups, and it is likely that the pattern of findings might differ if groups were better balanced in terms of their sample size. The small sample size of the always EHE group (*N* = 23) precluded any comparison with the group of children in EHE before the pandemic (*N* = 68); it is likely that children who never registered with a school (always in EHE) might differ from those who de-registered from school and opted for EHE at some point before the pandemic. Therefore, future research with a bigger sample size of families participating in EHE is needed to explore this and to replicate the pattern of findings. We used convenience sampling mostly through social media and EHE parent support groups, so it is very likely that the pattern of findings reflects possible sampling biases (e.g., people who took part in our study may have had positive experiences with EHE and the capacity in terms of time and resources to participate in an online survey).

## Conclusion

Our findings indicate that the main reason families of children with neurodevelopmental conditions such as autism and/or intellectual disability elected to de-register from school was the high level of needs that were not being met at school. COVID-19 had a more limited role in decisions to de-register and opt for EHE. Parents in our study reported that the schools did not provide individualised, flexible, and adapted education while they saw EHE’s main benefit as addressing these needs. EHE appeared to work well for families of children with neurodevelopmental conditions. While there was no evidence of better (or worse) child mental health in relation to the timing of de-registration or in comparison to school-registered children, concerns about the child’s mental health were an often-cited reason for de-registration and also a perceived benefit of home education.

## Data availability statement

Data collected during the course of the study have been made publicly available through the UK Data Service https://ukdataservice.ac.uk/ (database reference number SN 855596).

## Ethics statement

The studies involving human participants were reviewed and approved by the University College London Research Ethics Committee (Reference number: 20633/001). The patients/participants provided their written informed consent to participate in this study.

## Author contributions

The study was designed with input from authors VT, KG, RH, AA, GM, DH, and BT. LP led on the analyses and the first draft of the manuscript. AT and AK supported the analyses and write up. All authors guided the analyses, revised the manuscript, and agreed its final submitted version.

## Funding

Research was funded by the Economic and Social Research Council (UKRI Grant Ref: ES/W001993/1).

## Conflict of interest

The authors declare that the research was conducted in the absence of any commercial or financial relationships that could be construed as a potential conflict of interest.

## Publisher’s note

All claims expressed in this article are solely those of the authors and do not necessarily represent those of their affiliated organizations, or those of the publisher, the editors and the reviewers. Any product that may be evaluated in this article, or claim that may be made by its manufacturer, is not guaranteed or endorsed by the publisher.

## Supplementary material

The Supplementary material for this article can be found online at: https://www.frontiersin.org/articles/10.3389/fpsyg.2022.995217/full#supplementary-material
