# Supplementary material for: Elective home education of children with neurodevelopmental conditions before and after the COVID-19 pandemic started
Source: Front Psychol. 2022 Nov 10;13:995217. doi: 10.3389/fpsyg.2022.995217 (PMC9687365; doi:10.3389/fpsyg.2022.995217)
Supplement: Supplementary file 1 [file Table_1.docx]

# Supplementary material

# Supplementary material 1: Findings of the univariate associations between each potential covariate and each mental health outcome

Table 1: Pearson’s Correlations of demographic variables and DBC2 child anxiety total score

| **Demographic Variables** | **DBC2 anxiety score**  **R value** | ***P* value** |
| --- | --- | --- |
| Child age | -0.057 | 0.05 |
| Child is a boy | 0.122 | <0.001* |
| White ethnicity | -0.015 | 0.62 |
| Lives in England vs others | 0.047 | 0.11 |
| Child has additional physical conditions | 0.057 | 0.05 |
| Child has two or more NDCs | 0.098 | <0.001* |
| ID | -0.042 | 0.15 |
| SEND plan | -0.090 | 0.002* |
| Family socioeconomic deprivation | -0.019 | 0.52 |
| Parent disability | -0.18 | <0.001* |

NDCs=neurodevelopmental conditions; ID=intellectual disability; SEND= special educational needs and disabilities

Table 2: Pearson’s Correlations of demographic variables and child SDQ total internalising score

| **Demographic Variables** | **SDQ internalising score**  **R value** | ***P* value** |
| --- | --- | --- |
| Child age | 0.172 | <0.001* |
| Child is a boy | 0.063 | 0.0344* |
| White ethnicity | -0.027 | 0.37 |
| Lives in England vs others | 0.075 | 0.0110* |
| Child has additional physical conditions | 0.058 | 0.05 |
| Child has two or more NDCs | 0.061 | 0.04* |
| ID | -0.077 | 0.0097* |
| SEND plan | -0.119 | 0.0001* |
| Family socioeconomic deprivation | 0.013 | 0.67 |
| Parent disability | -0.127 | 0.001* |

NDCs=neurodevelopmental conditions; ID=intellectual disability; SEND= special educational needs and disabilities

Table 3: Pearson’s Correlations of demographic variables and child SDQ total externalising scores

| **Demographic Variables** | **SDQ externalising score**  **R value** | ***P* value** |
| --- | --- | --- |
| Child age | -0.194 | <0.001* |
| Child is a boy | -0.024 | 0.43 |
| White ethnicity | -0.006 | 0.85 |
| Lives in England vs others | 0.013 | 0.66 |
| Child has additional physical conditions | 0.046 | 0.12 |
| Child has two or more NDCs | 0.019 | 0.52 |
| ID | 0.076 | 0.0099* |
| SEND plan | 0.010 | 0.75 |
| Family socioeconomic deprivation | -0.006 | 0.83 |
| Parent disability | -0.085 | 0.01* |

NDCs=neurodevelopmental conditions; ID=intellectual disability; SEND= special educational needs and disabilities

# Supplementary material 2: Time of de-registration in two EHE groups

| **EHE pre-pandemic group**  **N=63** | | **EHE pandemic group**  **N=63** | |
| --- | --- | --- | --- |
| **Time** | **N (%)** | **Time** | **N (%)** |
| 2009 | 2 (3.3%) | March 2020 | 3 (4.8%) |
| 2011 | 1 (1.6%) | April 2020 | 4 (6.4 %) |
| 2013 | 3 (4.8%) | May 2020 | 5 (7.9%) |
| 2014 | 2 (3.2%) | June 2020 | 1 (1.6%) |
| 2015 | 3 (4.8%) | July 2020 | 3 (4.8%) |
| 2016 | 8 (12.9%) | August 2020 | 6 (9.5%) |
| 2017 | 5 (8.1%) | September 2020 | 16 (25.4%) |
| 2018 | 11 (17.7%) | October 2020 | 8 (12.7%) |
| 2019 | 20 (32.3%) | November 2020 | 2 (3.2%) |
| 2020 | 7 (11.3%) | December 2020 | 2 (3.2%) |
|  |  | January 2021 | 1 (1.6%) |
|  |  | February 2021 | 2 (3.2%) |
|  |  | March 2021 | 3 (4.8%) |
|  |  | May 2021 | 3 (4.8%) |
|  |  | June 2021 | 2 (3.2%) |
|  |  | July 2021 | 1 (1.6%) |
|  |  | September 2021 | 1 (1.6%) |

Note: EHE=elective home education.
